# Protected areas’ effectiveness under climate change: a latitudinal distribution projection of an endangered mountain ungulate along the Andes Range

**DOI:** 10.7717/peerj.5222

**Published:** 2018-07-12

**Authors:** Carlos Riquelme, Sergio A. Estay, Rodrigo López, Hernán Pastore, Mauricio Soto-Gamboa, Paulo Corti

**Affiliations:** 1 Programa de Magíster en Ecología Aplicada, Instituto de Ciencias Ambientales y Evolutivas, Facultad de Ciencias, Universidad Austral de Chile, Valdivia, Chile; 2 Laboratorio de Manejo y Conservación de Vida Silvestre, Instituto de Ciencia Animal y Programa de Investigación Aplicada en Fauna Silvestre, Facultad de Ciencias Veterinarias, Universidad Austral de Chile, Valdivia, Chile; 3 Instituto de Ciencias Ambientales y Evolutivas, Facultad de Ciencias, Universidad Austral de Chile, Valdivia, Chile; 4 Center of Applied Ecology and Sustainability (CAPES), Facultad de Ciencias Biológicas, Pontificia Universidad Católica de Chile, Santiago, Chile; 5 Aumen ONG, Coyhaique, Chile; 6 Dirección Regional Patagonia Norte, Administración de Parques Nacionales, San Carlos de Bariloche, Argentina

**Keywords:** Climate change, Ungulates, Protected areas, Endangered species, Species distribution models, Conservation

## Abstract

**Background:**

Climate change is one of the greatest threats to biodiversity, pushing species to shift their distribution ranges and making existing protected areas inadequate. Estimating species distribution and potential modifications under climate change are then necessary for adjusting conservation and management plans; this is especially true for endangered species. An example of this issue is the huemul (*Hippocamelus bisulcus*), an endemic endangered deer from the southern Andes Range, with less than 2,000 individuals. It is distributed in fragmented populations along a 2,000 km latitudinal gradient, in Chile and Argentina. Several threats have reduced its distribution to <50% of its former range.

**Methods:**

To estimate its potential distribution and protected areas effectiveness, we constructed a species distribution model using 2,813 huemul presence points throughout its whole distribution range, together with 19 bioclimatic layers and altitude information from Worldclim. Its current distribution was projected for years 2050 and 2070 using five different Global Climate Models estimated for scenarios representing two carbon Representative Concentration Routes (RCP)—RCP4.5 and RCP6.0.

**Results:**

Based on current huemul habitat variables, we estimated 91,617 km^2^ of suitable habitat. In future scenarios of climate change, there was a loss of suitable habitat due to altitudinal and latitudinal variation. Future projections showed a decrease of 59.86–60.26% for the year 2050 and 58.57–64.34% for the year 2070 according to RCP4.5 and RCP6.0, respectively. Protected areas only covered only 36.18% of the present distribution, 38.57–34.94% for the year 2050 and 30.79–31.94% for 2070 under climate change scenarios.

**Discussion:**

Modeling current and future huemul distributions should allow the establishment of priority conservation areas in which to focus efforts and funds, especially areas without official protection. In this way, we can improve management in areas heavily affected by climate change to help ensure the persistence of this deer and other species under similar circumstances worldwide.

## Introduction

Protected areas are one of the best tools for species and ecosystems conservation (Coetzee, 2017). However, because of climate change, some species, both animals and plants, will modify their distributions in response to new conditions, most often dispersing in search of more suitable environments (Bateman et al., 2016; Hovick et al., 2016). Under this scenario, many species would be pushed to change their distribution range and thus increase population fragmentation and habitat loss, in turn reducing survival and increasing extinction probability at local levels (Parmesan, 2006).

Disturbance of distribution ranges could also cause the existing network of protected areas (i.e., parks and reserves) to be less effective (Monzón, Moyer-Horner & Palamar, 2011). Currently, protected areas worldwide correspond to only 14.7% of Earth’s surface (19.8 million km^2^; UNEP-WCMC & IUCN, 2017), though the goal is to increase this to 17% by 2020 (CBD, 2010). Although protected areas have been described as an effective conservation planning tool because they contain greater species richness and abundance within their boundaries than non-protected areas, they do not necessarily offer similar protection when considering endemism of species within them (Gray et al., 2016).

Temperatures in southern South America are projected to increase at least 2 °C and precipitation decrease by 10–20% as an outcome of climate change by 2100 (Beaumont et al., 2011). These changes are predicted to lead to distribution shifts and possible extinctions of organisms dwelling in temperate forest and mountain (Bambach et al., 2013). Under this scenario, it is critical to estimate which areas would be suitable for species conservation, and for identifying climatic variables that will determine potential areas where they could survive (Olson & Dinerstein, 2002; Marchese, 2015).

One of the largest mammals of temperate forests of the southern Neotropical region is the huemul (*Hippocamelus bisulcus*), an endemic deer found in Chile and Argentina (Corti et al., 2011). This ungulate is distributed across 2,000 km in the southern part of the Andes mountain range (Vila et al., 2010). Here it is associated with sub-Antarctic forests dominated by beech tree species (*Nothofagus* spp.), low density understory, and periglacial areas (Vila et al., 2006). In addition, huemul is classified as Endangered by the International Union for the Conservation of Nature (IUCN), currently being the most threatened deer in the Neotropics (IUCN, 2016), where it is considered a flag and umbrella species throughout its distribution range (Povilitis, 1998; Quevedo et al., 2017). Identified threats to huemul include habitat loss and fragmentation, poaching, diseases and competition from domestic livestock, dog predation, and introduction of exotic species (Corti, Wittmer & Festa-Bianchet, 2010). Nowadays, it is estimated that there are less than 2,000 individuals and their distribution range has been reduced by 50% in relation to their historical range (Vila et al., 2006).

Because huemul is a late Pleistocene relic when cold and dry climatic conditions prevailed (Marín et al., 2013), current warming might be a serious threat for its persistence. Therefore, it is important to estimate probable negative effects of climate change on ecological projected parameters such as shifts in its future distribution range. Thus, our objectives were: (1) to estimate the current huemul distribution through a species distribution model (SDM), using climatic variables that could determine its presence; (2) to project current SDM into the future under different climate change scenarios; and (3) to estimate the efficiency of current protected areas in Chile and Argentina for both current huemul protection, and under projected climate change scenarios that potentially shift its distribution range.

## Materials & Methods

### Huemul occurrence area

**Figure 1:**
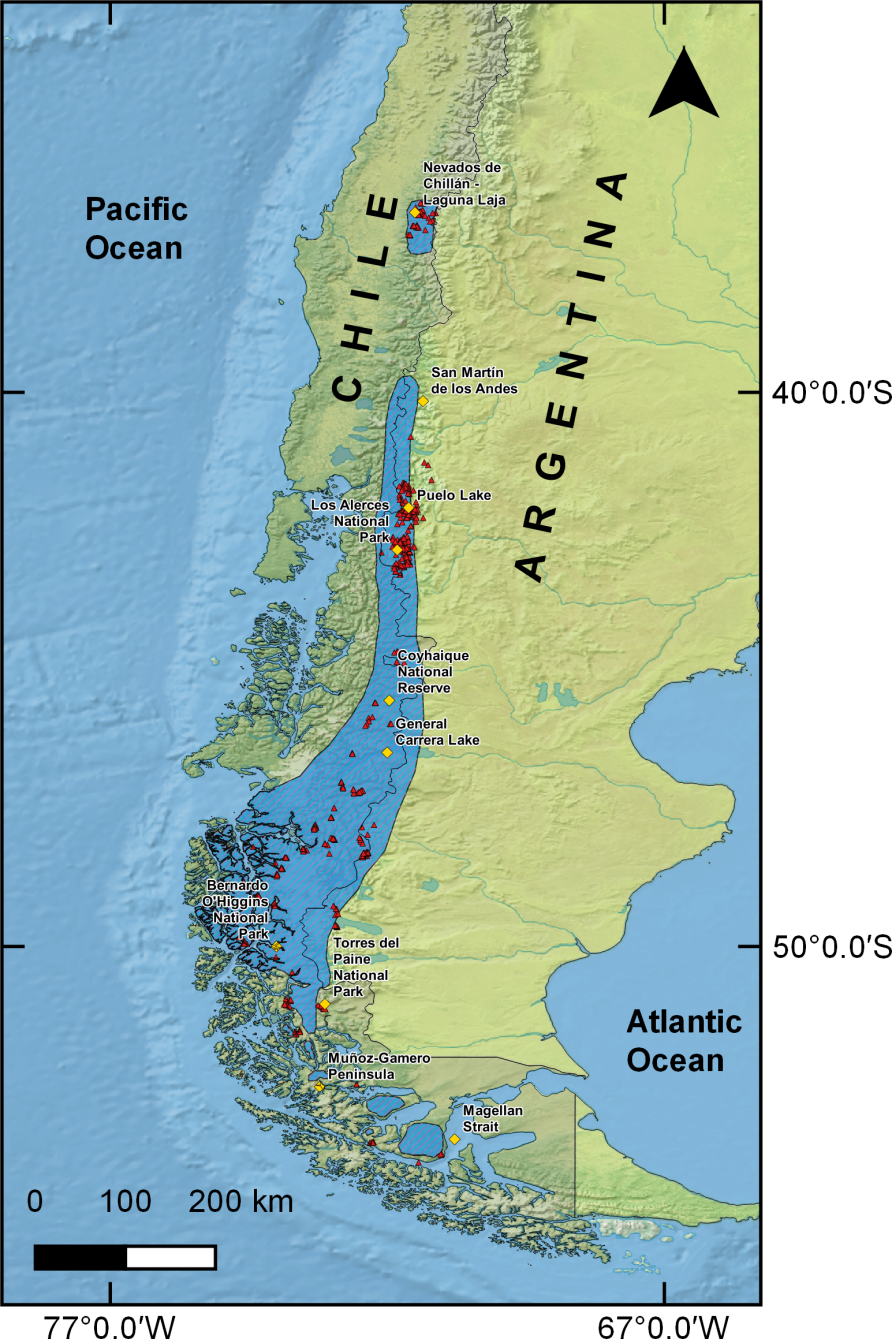
Huemul distribution and sample points. Current huemul distribution according to the International Union for Conservation Nature represented with diagonal lines (IUCN, 2016). Red triangles represent the huemul presence data used in this work and yellow squares represent relevant geographical landmarks along huemul distribution.

Presence records used to generate SDMs corresponded to 2,813 points of huemul occurrence collected throughout the whole known huemul distribution range, from Nevados de Chillán–Laguna del Laja (36°50′43″S & 71°23′56″W), to the Magellan Strait (53°28′51″S & 70°47′0″W; (Vila et al., 2006; Fig. 1). This database was obtained from available scientific literature (i.e., Corti et al., 2011; Marín et al., 2013; Quevedo et al., 2017) and from unpublished data collected between 2007 and 2016 from direct observation of animals and indirect records such as animal remains, tracks, and feces. To reduce oversampling biases in a particular area, we followed the recommendations of Fourcade et al. (2014), and restricted sampling to one presence record per km^2^, which resulted in a total of 856 effective points.

### Selection of environmental variables

Only climatic variables were used to build models because they give better projections for future scenarios than do land use variables (Martin et al., 2013; Titeux et al., 2017). In addition, vegetation variables are strongly related to the combination of temperature and precipitation, climatic variables, giving more useful habitat approximations for huemul (Quevedo et al., 2017). We felt that the use of multiple climatic proxies could, at least partially, overcome the lack of realistic vegetation predictions (Wilcox et al., 2017).

Environmental variables used to construct huemul distribution models were taken from Worldclim database (Hijmans et al., 2005), which contains information of 19 bioclimatic layers and altitude, in raster format, with an approximate resolution of one pixel per 1 km^2^. To avoid multicollinearity among variables, highly correlated ones were removed to reduce models error rates (Heikkinen et al., 2006). Multicollinearity was estimated through Pearson correlation coefficient (*r*_xy_), using the “VIRTUALSPECIES” package (Leroy et al., 2016) in R 3.4.0 (R Core Development Team, 2016). Those variables with a correlation coefficient *r*_xy_ ≥ 0.85 were removed. Variables kept after multicollinearity analysis were: altitude (ALT), annual mean temperature (BIO1), isothermality (BIO3), temperature seasonality (BIO4), minimum temperature of the coldest month (BIO6), annual temperature range (BIO7), mean temperature of the wettest quarter (BIO8), mean temperature of the driest quarter (BIO9), annual precipitation (BIO12), seasonality of precipitation (BIO15), and precipitation of coldest quarter (BIO19).

### Construction and evaluation of distribution models

To generate huemul distribution models we used the software MaxEnt 3.4.1k (Phillips, Dudík & Schapire, 2004; Phillips et al., 2017), which employs a machine learning process using the principle of maximum entropy, working only with presence data, and background (Phillips, Anderson & Schapire, 2006). Background is the representation of a potentially accessible area or one likely to be explored by the species (Peterson et al., 2011), and is used to contrast the information of presence points, allowing training of the model (Merow et al., 2016). To adjust the MaxEnt model, we created a training area through the software R 3.4.0 (R Core Development Team, 2016) with the package “ADEHABITAT” (Calenge, 2007). Then, a habitat use model was created through the Kernel Utilization Distribution method using all available huemul presence points (Moorcroft & Lewis, 2006). Within this background, we sampled 10,000 points to test against presence points in MaxEnt (Fourcade et al., 2014). Of 856 presences, MaxEnt only considered 619 points; using 434 (70%) of them to train our model, and 185 (30%) to evaluate it. The model was adjusted using 1,000 iterations, keeping its default characteristics. The result was expressed logistically, giving an approximation of habitat suitability with probability values ranging from 0 (not suitable) to 1 (ideal suitability; Peterson et al., 2011).

Evaluation results were based on the area under the ROC curve (AUC) and the regularized training gain. The ROC curve corresponds to the relationship between 1-specificity (false positive rate) and sensitivity (true positive rate; Phillips & Dudík, 2008). The AUC is the probability of correctly classifying the background presence points, with values ranging from 0.5 (explained by chance) to 1 (perfect discrimination between points of presence and background; Phillips, Anderson & Schapire, 2006). The evaluation of regularized training gain of each variable was performed using the Jack-knife method, where variables were individually assessed, generating a model with only one variable at a time, to estimate how much information a model delivered (Elith et al., 2011). Next, information contained exclusively by each variable was evaluated, calculating information loss and gain through the consecutive removal of variables, and then adjusting a model with the remaining variables (Phillips, Anderson & Schapire, 2006). Our model was replicated 10 times to improve its predictive capacity, so AUC and Jack-knife values corresponded to averages of those 10 replicates.

### Projection of the distribution model of huemul under two scenarios of climate change

To evaluate possible effects of climate change on huemul distribution, we modeled outcomes of current huemul distribution on layers of five future Global Climate Models (GCM) for the years 2050 (mean of years 2030–2060) and 2070 (mean of years 2060–2080). We selected these databases because GCM climatic projections could have regional variations (Yan et al., 2017). Databases were obtained from Worldclim and correspond to results of Phase 5 of Coupled Models Intercomparison (CMIP5). The GCM we used belong to: (1) CCSM4 (NCAR-UCAR, USA), (2) HadGEM2-ES (Met Office Hadley Centre, UK), (3) IPSL-CM5A-MR (Institute Pierre-Simon Laplace, France), (4) MRI-CGCM3 (Meteorological Research Institute, Japan), and (5) NorESM1-M (Norwegian Climate Centre, Norway). Climatic projection models are currently presented in four scenarios known as Representative Concentration Routes (RCP) that indicate the evolution of the range of greenhouse gas concentrations under different states of production of those gases (Moss et al., 2010). RCP scenarios were named in relation to their radiative value, which corresponds to the balance alteration between incoming and outgoing radiation from the atmosphere, causing changes in atmospheric constituents, as is the case of carbon dioxide (Moss et al., 2010). These scenarios have been projected until the year 2100, and have values ranging from 2.6 to 8.5 W/m^2^ (Van Vuuren et al., 2011a). From the four RCP available scenarios, we only used RCP4.5 and RCP6.0 for years 2050 and 2070. Scenarios RCP2.6 (the most optimistic; Stocker et al., 2013; Van Vuuren et al., 2011b) and RCP8.5 (the least optimistic; Riahi et al., 2011) would not be fulfilling the predictions, but current climatic variables are getting closer to values estimated by RCP4.5 and RCP2.6 models, showing an incremental increase in temperature from 2.0 to 4.9 °C (Raftery et al., 2017).

To predict possible huemul environment distribution shifts, either in relation to their current location or to suitable habitat, estimated models were converted to binary maps (0, not suitable; 1, suitable). Next, we used a threshold that maximizes sensitivity and specificity to balance commission and omission errors (Liu, White & Newell, 2013). Projections for years 2050 and 2070 GCM models were added to generate a concordance map with values ranging from 1 (where just one model estimated habitat suitability) to 5 (where all models match). Then, we used only the estimated concordance area of three GCMs. The resulting map of current estimated huemul distribution was compared to its distribution proposed by IUCN (2016). At the same time, a comparison was made between current distribution maps and the two future projections from climate change. All calculations were performed in the QGIS 2.12 (Quantum GIS Development Team, 2016).

### Evaluation of protected areas efficiency for huemul’s potential habitat

To evaluate the effectiveness of keeping huemul potential habitat within the protected areas system in Chile and Argentina, we estimated the overlap between calculated areas through the binary map and protected area systems of both countries. These projections corresponded to geographical information polygons, provided by the Chile’s National Forestry Service (CONAF) and by the Working Group on Protected Areas of the Ministry of Environment and Sustainable Development in Argentina. We also performed estimations of overlap in distribution models under the two projections of climate change (RPC4.5 and RCP6.0). Results of all calculations were presented in km^2^ and built in to the QGIS 2.12 (Quantum GIS Development Team, 2016).

## Results

### Current huemul distribution model

The model AUC was 0.96, indicating a high predictive value. Variables contributing the most in the model training were: seasonality of temperature (0.67), annual precipitation (0.59), and seasonality of precipitation (0.57). When analyzing the variables together, those ones containing the most information were annual precipitation (1.70), seasonality of precipitation (1.74), and precipitation of the coldest quarter (1.76; Table 1). The threshold that maximized sensitivity and specificity was 0.25, with which binary maps were generated.

**Table 1:** MaxEnt performance and environmental variables included in the model. Model performance and relative importance of each included variable according to Jack-knife analyses. For each variable used in the model, the first value corresponds to the gain of the adjusted model using only focal variables. The second value corresponds to the gain of the adjusted model using all but focal variables. The most important variables according to each criterion are bolded.

Environmental variables	Training gain with this variable	Training gain without this variable
Altitude (ALT)	0.06	1.78
**Temperature**		
Media annual (BIO1)	0.27	1.78
Isothermality (BIO3)	0.31	1.76
Seasonality (BIO4)	**0.67**	1.77
Minimum coldest month (BIO6)	0.20	1.79
Annual range (BIO7)	0.39	1.79
Media of wettest quarter (BIO8)	0.10	1.78
Media of driest quarter (BIO9)	0.31	1.77
**Precipitation**		
Annual (BIO12)	**0.59**	**1.70**
Seasonality (BIO15)	**0.57**	**1.74**
Coldest quarter (BIO19)	0.51	**1.76**

**Figure 2:**
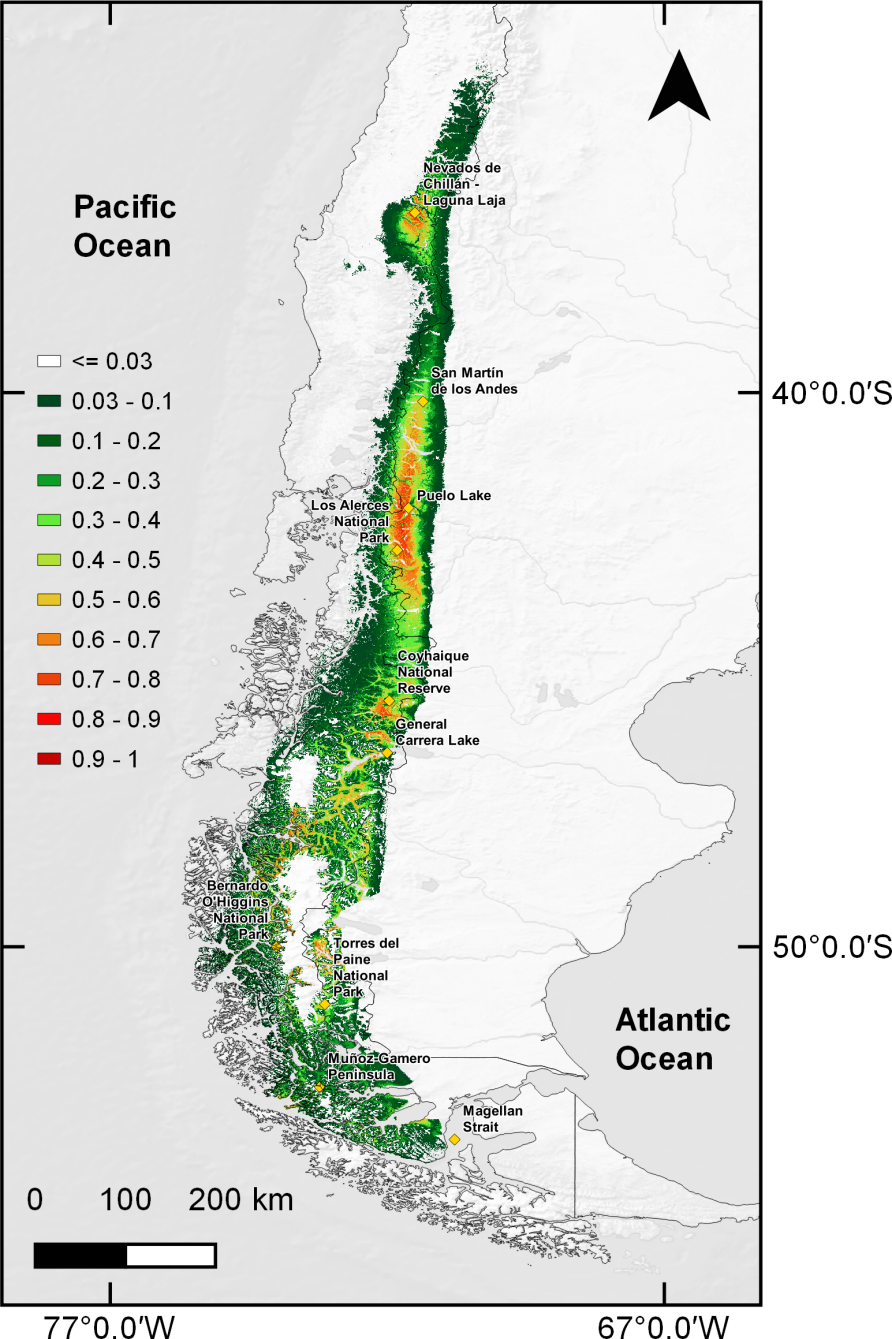
MaxEnt estimated huemul distribution. Huemul habitat current distribution model presented on a logistic scale of suitability. The suitability level is represented by a color gradient in the figure legend from white (not suitable) to dark red (most suitable). Yellow squares represent relevant geographical landmarks along huemul distribution.

The total calculated suitable area for huemul current distribution was 91,617 km^2^, mainly associated with the southern Andes Mountains (Fig. 2). In addition, it was possible to observe three large areas that contained the greatest habitat suitability: (1) an isolated area in Nevados de Chillán, (2) a large continuum running from San Martín de los Andes in Argentina (40°09′28″S & 71°21′12″W) and ending at General Carrera Lake (46°30′0″S & 72°00′0″W), and (3) an area expanding continuously southward and splitting into two branches, one following the eastern edge of the Andes into the Argentine side as far as Torres del Paine National Park in Chile, and the second running along the southern channels and fjords of Chilean coastal Patagonia through Bernardo O’Higgins National Park (51°2′20″S & 73°7′27″W).

### Huemul distribution under climate change projections

**Figure 3:**
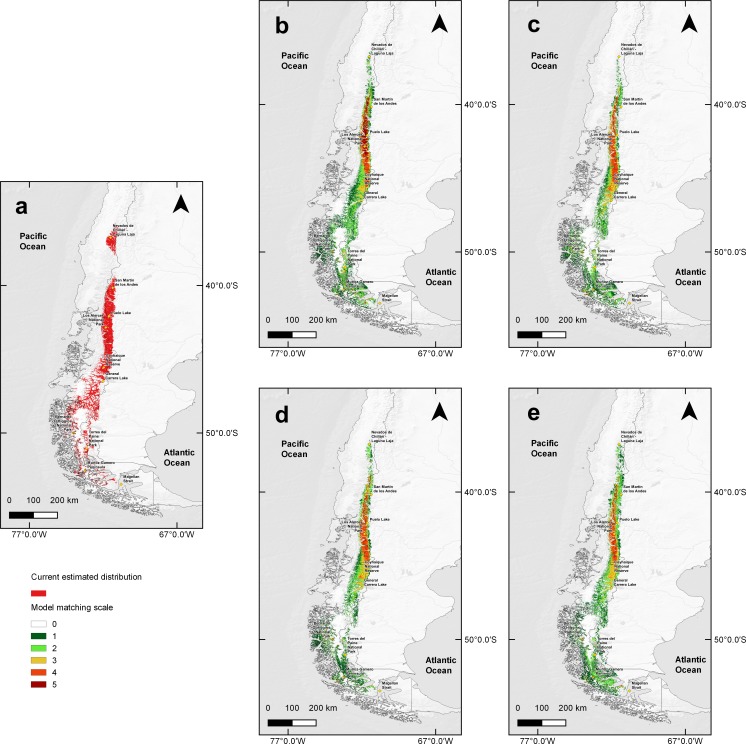
MaxEnt outcomes, current suitable habitat distribution and two projections scenarios of climate change. Comparison of current huemul distribution (A), and models generated from the combination of five Global Climate Models using the projections Routes of Representative Concentration (RCP), RCP 4.5, and RCP 6.0, RCP 4.5 projection for years 2050 (B) and 2070 (C). RCP 6.0 projection for years 2050 (D) and 2070 (E). Color scale indicates concordance levels among five generated distribution models, which go from dark green (one model match) to dark red (total models match). Yellow squares represent relevant geographical landmarks along huemul distribution.

Distribution models under climate change predicted a 59.86% habitat loss estimated in scenario RCP4.5 for year 2050, and a 58.57% loss for 2070. For the RCP6.0 scenario predicted a 60.26% reduction in the estimated area for 2050 and 64.34% loss for 2070 (Fig. 3). Along with these reductions in the area of suitable habitat, a southwards contraction in huemul distribution in all proposed scenarios was predicted. Reduction was most dramatic at Nevados de Chillán, where the suitable area almost completely disappeared (Fig. 3). In addition, under all scenarios, estimated distribution areas have a reduction in their eastern portions, but an expansion to west; this effect is more marked in projections for the year 2070 of the RCP4.5 and 6.0 models (Fig. 3).

### Estimation of the efficiency of protected areas for huemul conservation

**Figure 4:**
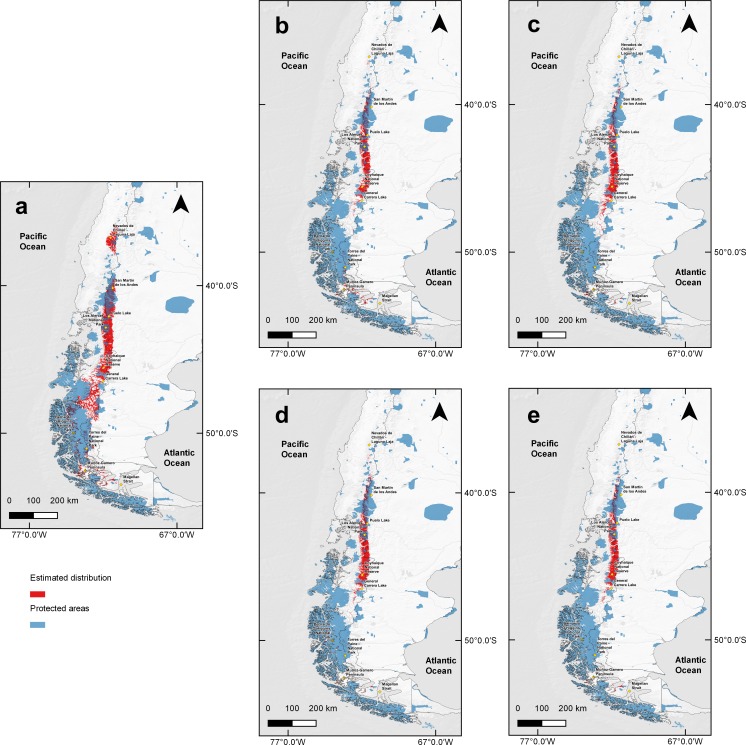
Binary map overlap with protected areas of Chile and Argentina, current and two projections of climate change. Protected areas cover of Chile and Argentina according to the current distribution model and five Global Climate Models combination under two scenarios of the climatic projections of the Representative Concentration Routes (RCP). Current estimation (A), with RCP 4.5 for years 2050 (B), 2070 (C) and RCP 6.0 for years 2050 (D) and 2070 (E). In red projected protected areas and in blue current protected areas for Chile and Argentina. Yellow squares represent relevant geographical landmarks along huemul distribution.

When comparing the estimated current huemul suitable habitat estimated distribution with that proposed by IUCN, there was a 40.16% (62,650 km^2^) concordance between them. However, when we overlapped current protected areas of Chile and Argentina on the model-projected area, only 36.18% was protected (Fig. 4). We found at least six large areas of high suitability that are currently not protected, and which are situated near towns, cities, and other human perturbed areas (i.e., cattle ranching and forestry plantations): (1) the area adjacent to Nevados de Chillán, (2) the area around San Martín de los Andes, (3) a large area beginning at the north end of Puelo Lake, south through the mountain range reaching the northern shore of General Carrera Lake, (4) the area located from the southern shore of General Carrera Lake to Bernardo O’Higgins National Park, (5) small areas located outside of Torres del Paine National Park, and (6) the last suitable area located on a group of islands near Muñoz-Gamero peninsula (52°32′0″S & 73°13′0″W).

**Figure 5:**
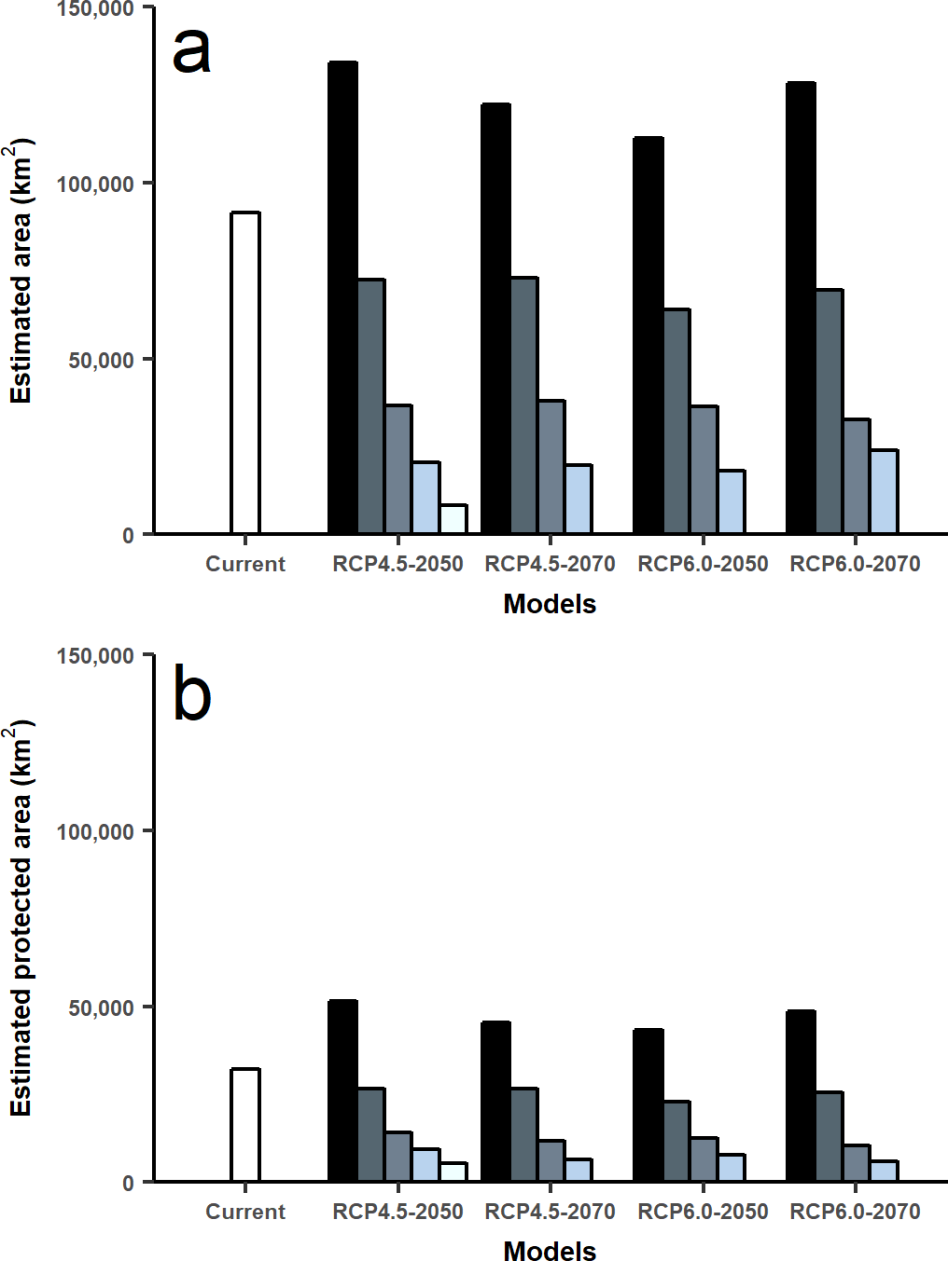
Total area estimated for each model and for protected areas. Calculated area (km^2^) for five distribution models considering the total projected surface and protected areas: amount of modeled area for current huemul distribution and concordant five GMC projections under two scenarios of climatic projections of RCP, RCP 4.5 and RCP 6.0 for years 2050 and 2070 (A). Amount of modeled area inside protected areas of Chile and Argentina. In white current distribution model and in grey scale the degree of concordance among five Global Climate Models (B).

Under climate change projections RCP4.5 and RCP6.0, protected areas would only cover 38.57% and 34.94% for 2050, and 30.79% and 31.94 for 2070 of huemul potential suitable habitat. This phenomenon is more intense if when comparing with the current total estimated area, where protection goes lower than 16% for the total potential huemul distribution (Figs. 4 and 5). In all future scenarios, the only remaining areas that would be unprotected were: (1) a small portion southward Nevados de Chillán, (2) one area western of San Martín de los Andes, (3) the area between Puelo and General Carrera Lakes, but more discontinuously than in current state, and (4) the area near Muñoz-Gamero peninsula.

## Discussion

### Current huemul distribution

The binary map of suitable huemul habitat distribution agreed in almost 50% with the one IUCN (2016) estimated. However, our current distribution model corresponded to historical distribution boundaries described for huemul, ranging from Cachapoal River (34°10′0″S) in the north to Brunswick Peninsula at the southern limit (53°52′0″S, Vila et al., 2006). Areas of highest suitability for huemul presence were always in the southern Andes Mountains, which agrees with Quevedo et al. (2017) findings for the Valdivian Ecoregion, and with Vila et al. (2006) for the total distribution. This mountain range is dominated by tree species of genus *Nothofagus* spp. (Donoso, 1987), which seem a fundamental component for huemul diet and shelter (Vila et al., 2010). Current discrepancy of protected areas with estimated huemul distribution has also been recorded in other Neotropical ungulates, such as pudu (*Pudu puda;* Pavez-Fox & Estay, 2016), taruka (*Hippocamelus antisensis;* Mata et al., 2018), and guanaco (*Lama guanicoe;* Castillo et al., 2018). Thus, the findings of this research are important to improve the efficiency of conservation areas for species under similar circumstances of huemul, prioritizing sites suitable for animals with the potential of being resilient to climate change (Pringle, 2017).

The total calculated suitable habitat area was subdivided into three main sectors: (1) Nevados de Chillán, (2) North Patagonia (centered on Puelo Lake), and (3) South Patagonia, including the Chilean fjords. This subdivision was consistent with huemul phylogeographic information (Marín et al., 2013), where a similar distribution pattern was obtained of three geographically separated populations. Huemul’s current distribution is most probably an outcome of the Last Maximum Glacier (Marín et al., 2013). Woolbright et al. (2014) indicated that species surviving glacial events, subsequently expanded from nucleus areas or refugia, but because of changes in environmental conditions, they fragmented their distribution after their expansion. This has also been described in other ungulates (i.e., mountain goats *Oreammos americanus*; Shafer, Côté & Coltman, 2011), resulting in isolated of their populations and a consequential reduction in genetic diversity, and has been described for huemul (Corti et al., 2011).

### Climatic variables influence on huemul habitat suitability

Among variables used in the models to predict the most suitable habitat areas, those giving the most information for model construction were: seasonality of temperature, annual precipitation, seasonality of precipitation, and precipitation during the coldest quarter. Annual precipitation is positively correlated with above ground net primary production at regional levels (Hsu, Powell & Adler, 2012), which could explain the mosaic of plant communities in the Patagonian region. Here water availability is limited by landscape topography, generating a vegetation gradient from forest, shrub lands, and steppe dry areas (Paruelo et al., 1998; Paruelo, Jobbágy & Sala, 2001). On the other hand, seasonality of precipitation would act as a limiting factor for vegetation growth (Hsu, Powell & Adler, 2012), since variation in water availability affects vegetation, shaping the amount of environmental energy, and limiting available habitats (Hawkins et al., 2003). Although precipitation during coldest quarter seemed to have no effect on primary production (Cramer & Hoffman, 2015), the winter season does have an important effect on several other organisms (Williams, Henry & Sinclair, 2015), decreasing survivorship in some cases (Brodie et al., 2013). Seasonality of temperature could indicate that habitats used by huemul can withstand a moderate variation of seasonal temperature, influencing tolerance of vegetation associated with this deer. A similar effect has been observed for African savannah vegetation, where the effect of variation in warm seasons reduced vegetation, producing a reduction in abundance and changes in distribution of ungulate populations inhabiting them (Ogutu & Owen-Smith, 2003).

### Estimation of climate change effect

The predicted reduction in suitable habitat for huemul (ca. 56–64%) due to climate change is higher than averages estimated for other South American mammals (e.g., a 37% reduction of habitat in 87% of analyzed species; Schloss, Nunez & Lawler, 2012). In addition to a reduction in range extension of huemul’s possible habitats, we observed latitudinal and altitudinal variation of suitable habitat for this deer. This pattern of alteration in wildlife distribution has been proposed globally, because species are expected to be able to adapt to future environmental changes with regard to latitudinal and altitudinal migrations (Chen et al., 2011). Mountain goats are an example of this habitat distribution shift, with altitudinal variation of their habitat use expected in their response to climate change (White, Gregovich & Levi, 2017). Similar habitat variation is expected for ibex (*Capra ibex*), chamois (*Rupicapra rupicapra*), and red deer (*Cervus elaphus*; Büntgen et al., 2017). Although for southern hemisphere ungulates there is a lack of similar information, our results for huemul suggest comparable patterns of habitat variation. Schloss, Nunez & Lawler (2012) suggested that sources of habitat variations from climate change would affect most mammal species throughout the Americas, due to limitations in their dispersal capacities. An average of 9.2% of mammal species are expected to be unable to adjust to habitat variation, with as many as 39% in some regions of the American continent. In our case, the possibility of huemul migration could be limited due to its low dispersal capacity (8 km; Gill et al., 2008), its high site fidelity, its low population density, and its frequent population isolation and fragmentation (Corti, Wittmer & Festa-Bianchet, 2010; Corti et  al.,  2011).

### Protected areas effectiveness: present and future

The current distribution model showed that there is suitable habitat for the huemul in 69 protected areas of Chile and Argentina. These are mainly found in the foothills of the southern Andes and fjords of western coastal Patagonia. However, these areas protect only 36.18% of the total estimated huemul distribution area, and thus are inadequate if we consider all the threats that this deer faces within and outside protected areas (Corti, Wittmer & Festa-Bianchet, 2010). The area between Los Alerces National Park and General Carrera Lake has a low level of protection, which is of special concern considering that it represents an important area for huemul persistence. It represents the biogeographic limit between two clades (Marín et al., 2013) where a possible genetic exchange between populations might occur.

The lack in conservation areas is a global problem. Of 85% of the world’s threatened vertebrates only 17% occur in any conservation area (Venter et al., 2014). A similar picture is found for huemul where current protected areas are inadequate. Originally though, the current protected areas were based on guidelines available at the time (Armesto et al., 1998), but unfortunately these guidelines no longer meet requirements for huemul conservation. In addition, animals might use less suitable habitats when human disturbance is reduced (Laliberte & Ripple, 2004). Thus, some lower suitability areas, particularly forest plantations, have shown that they can be favorable environments for huemul as long they are subject to specific management conditions (Sandvig et al., 2016).

Conservation of flag and umbrella species like huemul, that are predicted to be affected by climate change, could serve broader conservation actions, by protecting highly vulnerable environments or ecosystems associated with them (Forrest et al., 2012). The conservation of huemul and its habitat will help protect ecosystems associated with the southern Andes, mainly the Valdivian Ecoregion,–an important biodiversity hotspot (Olson & Dinerstein, 2002; Lara et al., 2009).

## Conclusions

To address huemul conservation and probable effects of climate change, conservation recommendations should focus on areas that currently have no or a low degree of protection, but which have a high potential for suitable huemul habitat in the future. For example, areas between Puelo and General Carrera Lakes, and the western portion of Muñoz-Gamero peninsula are a priority because they are sites predicted by our model to be less affected by climate change in terms of their suitability for this deer.

It is also advisable to establish management strategies for areas of lower suitability where there are current known huemul populations, and in areas contiguous with them to allow connectivity among populations. Areas like Nevados de Chillán, for example, could be strongly impacted by climate change, so the protection of lower quality habitat in this region will allow huemul movements, which will probably be crucial for the persistence of this isolated population.

The future of conservation areas worldwide will face climate change challenges, where resilience of conservation sites to these effects will be crucial for the management of endangered species (Pringle, 2017). Thus, the identification of those resilient sites plus buffer zones around those protected areas will be a priority in order to promote connectivity between them (Xun, Yu & Wang, 2017), or even modify productive area management strategies to make them suitable for wildlife.

##  Supplemental Information

10.7717/peerj.5222/supp-1 Supplemental Information 1 R script to create the shape from cut rasters to train Maxent

## Additional Information and Declarations

### Data Availability

The following information was supplied regarding data availability: R script to create the shape from cut rasters to train MaxEnt is uploaded as Supplemental File. Huemul deer data is available in the cited references and governmental agencies of Chile and Argentina. However, some data is restricted because they belong to private forestry companies. In addition, huemul is critically endangered deer and uploading the specific location of this deer could compromise the species due to poaching. Thus, for ethical reasons, the data cannot be freely accessed.
